# High-throughput sequencing of the T-cell receptor repertoire: pitfalls and opportunities

**DOI:** 10.1093/bib/bbw138

**Published:** 2017-01-10

**Authors:** James M Heather, Mazlina Ismail, Theres Oakes, Benny Chain

**Affiliations:** 1 Massachusetts General Hospital, Boston, MA; 2 University College of London, Bloomsbury, UK; 3 Division of Infection and Immunity, University College of London, Bloomsbury, UK

**Keywords:** T-cell receptor, high-throughput sequencing, immune repertoire, somatic recombination, generation of diversity, personalized biomarkers

## Abstract

T-cell specificity is determined by the T-cell receptor, a heterodimeric protein coded for by an extremely diverse set of genes produced by imprecise somatic gene recombination. Massively parallel high-throughput sequencing allows millions of different T-cell receptor genes to be characterized from a single sample of blood or tissue. However, the extraordinary heterogeneity of the immune repertoire poses significant challenges for subsequent analysis of the data. We outline the major steps in processing of repertoire data, considering low-level processing of raw sequence files and high-level algorithms, which seek to extract biological or pathological information. The latest generation of bioinformatics tools allows millions of DNA sequences to be accurately and rapidly assigned to their respective variable V and J gene segments, and to reconstruct an almost error-free representation of the non-templated additions and deletions that occur. High-level processing can measure the diversity of the repertoire in different samples, quantify V and J usage and identify private and public T-cell receptors. Finally, we discuss the major challenge of linking T-cell receptor sequence to function, and specifically to antigen recognition. Sophisticated machine learning algorithms are being developed that can combine the paradoxical degeneracy and cross-reactivity of individual T-cell receptors with the specificity of the overall T-cell immune response. Computational analysis will provide the key to unlock the potential of the T-cell receptor repertoire to give insight into the fundamental biology of the adaptive immune system and to provide powerful biomarkers of disease.

## Introduction

**Figure 1:**
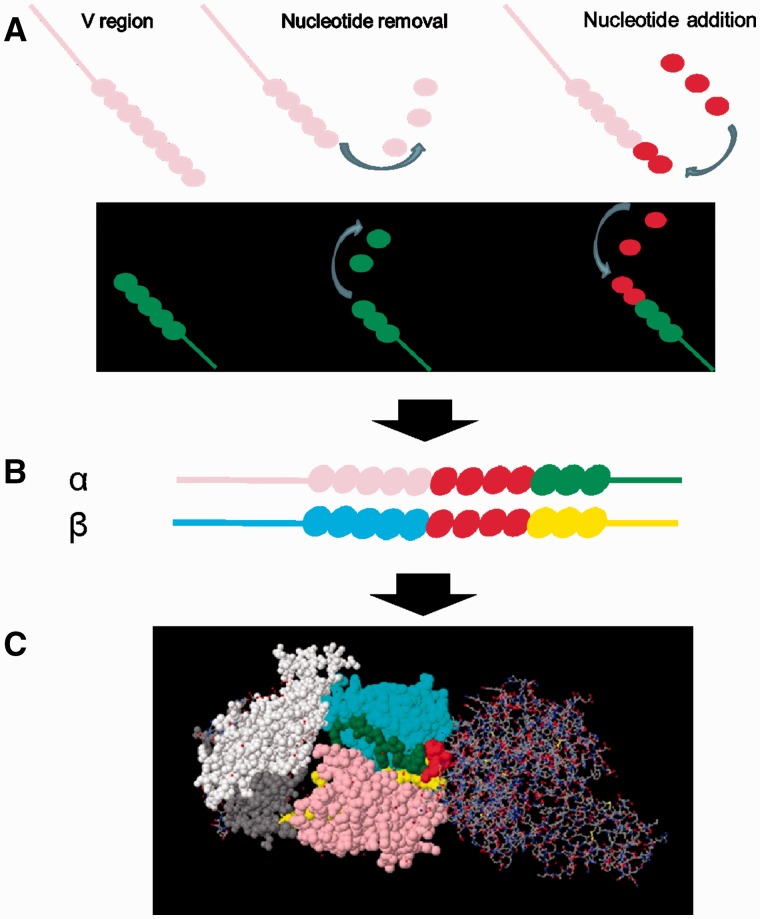
T-cell recombination and the generation of diversity. Individual V and J genes are selected stochastically (but not uniformly) and recombined during T-cell development in the thymus. During recombination base pairs can be removed and/or added at the junction before the final ligation (**A**). Both alpha and beta genes undergo recombination independently. Beta genes incorporate an additional D region minigene between V and J, giving rise to two junctions (not shown). Finally, alpha and beta V regions are transcribed, spliced onto their respective constant regions (**B**) and translated, and the two proteins heterodimerize to give rise to a single TCR (**C**). The TCR/MHC/peptide complex shown here is derived from the PDB structure 1FYT, and displayed using RasMol [3]. The TCR is shown in space fill, and the peptide/MHC complex is shown in stick representation on the right. Pink – Vα; yellow – Jα; blue – Vβ; green – Jβ; and red – CDR3.

The adaptive immune system of jawed vertebrates uses imprecise somatic DNA recombination to generate a rich and diverse array of antigen-specific receptors on B cells (BCR) and T-cells (TCR). The mechanism for the generation of variable antigen receptor diversity has been studied in great detail (e.g. see reviews [1, 2], and diagrammatic illustration in Figure 1). In brief, the locus encoding each chain is comprised of multiple gene segments (‘minigenes’), which include V, D and J genes. Recombination of the genomic DNA during lymphocyte development results in the physical joining of one of each available type of gene segment, and excision of the intervening DNA. The combinatorial diversity generated by there being multiple V, J and (in certain chains) D genes to ‘choose’ from is hugely augmented by non-templated nucleotide additions and deletions occurring at the junctions of the segments, during the joining process. This system produces an enormous diversity of receptor sequences [4, 5], and each biological sample of blood or tissue will typically have thousands or millions of receptors. This prevented a global analysis of the full repertoire of B- or T-cell antigen receptors using conventional DNA sequencing.

However, the rapid advances in high-throughput DNA sequencing (HTS) over past decades [6] have opened the way for increasingly robust and extensive BCR and TCR repertoire studies. The extraordinary heterogeneity of the immune repertoire poses significant challenges not just for the laboratory sequencing pipeline but also for the subsequent analysis of the genomic data sets that are generated. In this review, we focus specifically on the computational and bioinformatic analysis of the TCR repertoire. We outline the major steps in processing of repertoire data, considering low-level processing of raw sequence files and high-level algorithms that seek to extract biological or pathological information from the data. We survey some of the tools that have been developed for such analyses and indicate the potential of repertoire analysis to give insight into the fundamental biology of the adaptive immune system and to provide powerful biomarkers of disease [5, 7–9].

The TCR repertoire can be regarded as an ultimate example of a high-dimensional and intimately personalized biomarker, the hallmarks of precision medicine of the future. The first decade of high-throughput TCR sequencing analysis has focused, predictably, on the challenging but technical problems of gene assignment and error correction. As the technology becomes more established and robust, attention is turning toward the development of the sophisticated computational tools which are required to make sense of these novel but often impenetrable indicators of immune function.

## The processing pipeline: an overview

**Figure 2:**
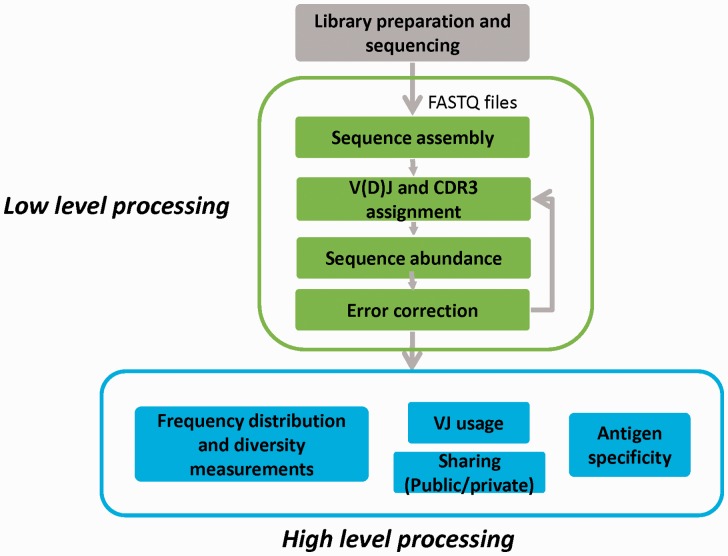
The main stages involved in the study of immune repertoires. Grey box (top): library preparation and sequencing. Green boxes (middle): low-level processing that includes sequence assembly, assignment to genomic V, D and J genes, extraction of CDR3 regions and error correction. See sections ‘Gene assignment’, ‘Sequence and abundance error-correction strategies’ and ‘Benchmarking and its challenges’ of this review. Blue boxes (bottom): high-level processing and analysis, which includes diversity measurements, and determining clonal frequency distributions, analysis of differential V and J usage, analysis of inter-individual sharing of TCR sequences (public versus private) and relationship between sequence and antigen specificity. See sections ‘High-level repertoire processing: revealing biological and clinical meaning’, ‘Measures of diversity’ and ‘Antigen specificity’ of this review.

The main stages involved in the study of immune repertoires are illustrated in Figure 2. They can be broadly divided into library preparation and sequencing, low-level processing, which includes determining the receptor sequences and assigning them to genomic V, D and J genes, and high-level processing and analysis.

Although we focus on *in silico* analysis, a brief survey of the main methods currently used for TCR repertoire library preparation and sequencing is helpful. High-throughput TCR library preparation is reviewed in detail elsewhere [10–13]. A number of sequencing technologies have been applied to studying TCR repertoires, but as with genomics and transcriptomics at large, the Illumina platform has become the *de facto* standard ([14], http://www.illumina.com/). This position has been achieved as a result of dramatic reductions in per base sequencing costs, and huge improvements in read length and quality. However, several pipelines exist for processing of biological samples before sequencing. The objective of all pipelines is to document as completely as possible the rearranged alpha and beta TCR gene sequences and determine their abundance within a sample. The main commercial service provider of TCR sequencing amplifies from genomic DNA or messenger RNA (mRNA) ([5], http://www.adaptivebiotech.com/immunoseq). Although most details of their pipeline are proprietary, the methods are based on amplifying recombined TCR genes using a multiplex polymerase chain reaction (PCR), with a set of primers that can capture all possible combinations of V and J genes [5]. Non-recombined genes are not amplified because of the large introns between V and J genes. In contrast, several research groups have developed RNA/complementary DNA (cDNA)-based techniques for repertoire analysis. Using RNA as starting material, rapid amplification of complementary DNA (cDNA) ends (RACE) technologies can be applied [15, 16], thus decreasing the amount of PCR bias that may result from different efficiencies of primers for different V and J genes. Starting with DNA has a number of advantages in terms of ease of sample collection and stability of storage. Furthermore, DNA-based strategies are not influenced by heterogeneity in mRNA transcription or stability. In contrast, a major benefit of RNA-based techniques is that they are easily adapted to allow the introduction of unique molecular identifiers (UMIs), which can provide accurate quantitative estimates of TCR abundance in a sample. As discussed in detail below, the intrinsic heterogeneity of PCR means that this is essential to achieve a robust quantitation of the repertoire.

Once TCR genes have been enriched, amplified and sequenced, the raw sequence data files (which are typically output in FASTQ format) need to be processed to yield meaningful biological information. In common with genomic or RNA-seq protocols, the first stage of processing is to match the sequence to the known genomic reference, in this case assigning each TCR to its germline component gene sections (Figure 2, low-level processing). This process is, however, much more difficult for TCRs, both because the TCR locus is made up of many similar V, J, and (in some cases) D genes, which must be distinguished accurately, and also because these recombined regions will also contain deletions and non-templated additions introduced during the recombination process (Figure 1). Several approaches to gene assignment are discussed in the ‘Gene assignment’ section below. Considerable effort has been put into developing algorithms which correct the resulting nucleotide sequences for PCR and sequencing errors, and which correct for biased PCR amplification. These are discussed in the sections ‘Sequence and abundance error-correction strategies’ and ‘Benchmarking and its challenges’.

Finally, once a corrected set of assigned sequences has been assembled (‘a repertoire’), approaches to mining these data sets (categorizing and comparing different repertories, measuring diversity, annotating TCR antigen specificity, etc.) and extracting biological or pathological meaning can then be explored (Figure 2, high-level processing). The approaches to high-level processing of TCR repertories are discussed in the sections ‘High-level repertoire processing: revealing biological and clinical meaning’, ‘Measures of diversity’ and ‘Antigen specificity’ below.

## Gene assignment

In the past 5 years, a considerable number of low-level processing software programs have been published. Often, the software has been developed alongside experimental library preparation pipelines, and are primarily designed to work on the data produced by this particular pipeline. We have compiled a list of all the open source software tools for low-level processing of TCR repertoire sequence data of which we are aware (Table 1). Some of the special features of these different packages are discussed in the paragraphs below. Tools for analysis of TCR sequences within global transcriptomic RNA-seq data, including single-cell RNA-seq data, are discussed separately.

**Table 1:** A comparison of the features of open source programs available for processing of HTS TCR sequences^a^

	**Analysis tool name**^b^	Input format	Availability (online/stand-alone)	Maximum input size (number of sequences)	Filtering/error-correction steps built into analysis pipeline			
					Sequence read quality	**Unique identifier**^c^	**Clustering**^d^	**Frequency**^e^
1	IMGT/V-QUEST [17]	FASTA	Online	50	No	No	No	No
2	IMGT/HighV-QUEST [18]	FASTA	Online—account log in required	150 000	No	No	Yes	No
3	IgBLAST [19]	FASTA	Online/stand-alone (command line, C ++)	<1000 (online)/none (stand-alone)	No	No	No	No
4	Decombinator [20]	FASTQ	Stand-alone (command line, Python)	None	Yes (barcode quality)	Yes	Yes (barcode clustering)	No
5	Vidjil [21]	FASTA/FASTQ	Stand-alone (command line, C ++)	None	No	No	Yes	Yes
6	MiXCR (+ MiGEC) [22]	FASTA/FASTQ	Stand-alone (command line, Java)	None	Yes	Yes	Yes	Yes
7	IMonitor [23]	FASTA/FASTQ	Stand-alone (command line, Perl, R)	None	Yes	No	Yes	No
8	IMSEQ [24]	FASTA/FASTQ	Stand-alone (command line, C ++)	None	Yes	No	Yes	No
9	LymAnalyzer [25]	FASTQ	Stand-alone (command line/GUI, Java)	None	No	No	Yes	No
10	TCRklass [26]	FASTQ	Standalone (command-line, C ++/Perl)	None	Yes	No	No	No
11	Recover TCR (RTCR) [27]	FASTQ	Stand-alone (command line, Python)	None	Yes	Yes	Yes	No
12	TRIg [28]	FASTA	Stand-alone (command line, Perl)	None	No	No	No	No

^a^Programs listed do not include tools for single-cell analysis, which are mentioned in text.

^b^Full link to tool Web page:

1, 2. http://www.imgt.org

3. https://www.ncbi.nlm.nih.gov/igblast/

4. https://innate2adaptive.github.io/Decombinator/

5. http://www.vidjil.org

6. https://github.com/milaboratory/mixcr

7. https://github.com/zhangwei2015/IMonitor

8. http://www.imtools.org

9. https://sourceforge.net/projects/lymanalyzer/

10. https://sourceforge.net/projects/tcrklass/

11. https://github.com/uubram/RTCR

12. https://github.com/TLlab/trig

^c^Molecular barcoding.

^d^Combining clones that are similar (according to a distance threshold).

^e^Removal of clones occurring less than a frequency threshold.

The International Immunogenetics Information System, or IMGT, was first developed in 1989 as a centralized repository for immunogenetics-related data ([29], www.imgt.org). It remains the most widely used reference point for immunogenetics, and specifically for maintaining the IMGT/GENE-DB database [30], which contains a well-annotated set of genomic V, D and J genes from several species (including mouse, rat, rabbit and man). This set of genes provides the reference for most TCR gene assignment tools. An important role of the IMGT has been to standardize the nomenclature for TCR gene segments [31], although it should be noted that older nomenclatures are commonplace in the literature even today, and can cause considerable confusion in repertoire analysis. A useful comparison between the different nomenclatures can be found at www.imgt.org/IMGTrepertoire/LocusGenes/#J.

In addition to providing the reference sequences for TCR genes, the IMGT resource page also provides various tools for sequence assignment, including IMGT/V-QUEST [17, 32], and a higher-throughput version, IMGT/HighV-QUEST [18, 33]. Both versions are accessible via Web portals. IMGT/V-QUEST allows up to 150 000 sequences to be submitted in one session, but this will still often not be sufficient to cope with the tens or hundreds of millions of reads produced by typical HTS experiments. Gene assignment uses global pairwise alignment algorithms to identify the best fitting V, D and J genes, and then the Smith–Waterman local alignment algorithm to determine the deletions at the end of the V(D)J genes, and the insertions between them. These algorithms are relatively slow, limiting the use of these tools for HTS analysis.

Faster versions of similar global alignment algorithms have been developed specifically for TCR HTS using the same principles of aligning to germline sequences. IgBLAST was first developed as a tool for the analysis of immunoglobulin sequences, but has since added an option for TCR sequence analysis [19] and uses the BLAST algorithm, a local alignment method, to search query sequences against germline sequences in the IMGT and NCBI databases. Similarly, IMonitor [23] also uses BLAST and additionally has a second alignment step that finds exact matches in the non-CDR3 sequences. Recover TCR, or RTCR [27], uses Bowtie2 [34] as its default alignment module, which allows either local or end-to-end alignment. An additional layer of complexity in TCR gene assignment is that mRNA from T-cells frequently contains sequences that do not follow the classical rules of VDJ recombination, but may include partial recombination events, retained intergenic sequences or concatenated J genes [35]. The analysis of these nonregular sequences is addressed directly by TRIg [28], which tests alignment with the whole TCR locus, and not just exons.

IMmunogenetic SEQuence Analysis (IMSEQ) [36] also characterizes TCR sequences by aligning the input to the germline, and additionally uses a checking step, which looks for flanking V and J regions as well as the CDR3 region. The algorithm starts by finding matches in short core sequences before extending the alignment for sequences that have an error score below a certain threshold.

An alternative strategy for gene assignment is the use of partial sequences, or tags, to identify specific V and J regions. Decombinator [20] implements a modified Aho-Corasick [37] search algorithm to find such matching strings (or tags with a single mismatch), which achieves much faster gene assignments. Once the V and J region are identified, alignment with the actual sequence identifies the end of the gene segment region and, hence, identifies insertions and deletions. Apart from speed, the tag strategy is not sensitive to errors in V or J regions outside the short tags themselves. Potential errors are, therefore, confined to the CDR3 region (see below). An updated version of Decombinator, together with a full list of V and J sequences for human and mouse, and the list of identifier tags, is available at https://github.com/innate2adaptive/Decombinator. The Vidjil algorithm [21] uses a heuristic method, using unique substrings in the germline genes to assign V and J genes. Using the substrings, it specifies a ‘window’, which is centered on the sequences between the V and J, and is large enough to include both regions. Identical windows (provided no sequencing errors) are clustered and counted, representing a clonotype and its respective abundance. Further, VJ refinement is carried out, and Vidjil outputs the top 20 most abundant clones for further analysis. Another analysis tool called LymAnalyzer [25] implements a different strategy using short sequence tags for gene assignment. Alignment to the reference sequence (by default taken from the IMGT database, although users may specify a different reference) is carried out by identifying short continuous sets of sequences, or tags, that are most closely related to a reference sequence in the database. The algorithm allows for mismatches by moving along subsequent tags in the instance where exact matching could not be found in the first tag (assigned starting from the 3′ end for the reference V gene, and 5′ for the J gene). The sequences are then translated to CDR3 sequences and clustered. Identical sequences are grouped together and counted to represent clonotype frequency, where groups are classified as ‘core’ or ‘minimum’ sequences. Each of the sequences in the ‘minimum’ group is checked against the ‘core’ sequences using a distance measure called the Hamming distance, and if sequences meet a specific threshold (default = 2) they are merged with the closest ‘core’ group. TCRklass [26] offers another variant of partial sequence matching, using K-strings matching to select the J or V gene from a given reference set, which gives the best match to a query sequence.

MiTCR [38] and its successor MiXCR [22] combine the use of tags with more classical alignment tools. First, subsequences, or seeds, that match conserved start and end patterns in the V and J regions are identified. The alignment of the subsequences are then extended and given scores. The output is the top-scoring alignment, taking into account the position of conserved residues. Alignments containing more than a user-specified threshold of mismatched nucleotides are discarded. MiXCR also provides an assignment to specific D regions. However, D regions are short and similar to each other making accurate assignment difficult, and most studies of TCR repertoire simply include the D region within the CDR3 sequence.

The reliable and unambiguous assignment of V and J genes only requires in the order of 150 base pairs (bp) 5′ to the start of the constant region. As most TCR sequencing pipelines produce amplicons, which are specifically targeted to this region of the TCR, the read length of modern HTS machines (often >150 bp paired end reads) means that sequence assembly is rarely needed before gene assignment. However, there are exceptions where TCR gene regions are enriched from randomly fragmented DNA using V and J region baits [39] or alternatively are recovered from total RNA-seq data [40–42]. A particularly important example of the latter is the application of single-cell RNA-seq, which allows matching of alpha and beta chains (and hence potentially recovering antigen specificity) and simultaneous expression profiling of T-cell functional states [41, 43, 44]. The specialized bioinformatic tools required for an assembly of TCRs from RNA-seq data are described in the studies referenced above.

In addition to V(D)J assignment, TCR repertoire analysis typically requires identification and translation of the hypervariable CDR3 sequence, which is believed to play the key role in determining antigen specificity [45] (Figure 2). Translation from nucleotide to amino acid sequence itself is relatively straightforward, using readily available packages and functions existing in most popular scripting languages, e.g. using Biopython in Python [46] or Biostrings in R [47]. Translated sequences can then be scanned for conserved amino acid motifs defining the CDR3 region. IMGT defines CDR3 regions to run inclusively from the second conserved cysteine residue in the 3′ end of the V gene to the phenylalanine residue in the conserved FGXG motif found in the J gene [48]. Although the second conserved cysteine residue is usually the last C-terminal cysteine in a V gene, there are some genes in which it is not, and cysteines can be produced (albeit rarely) during the stochastic recombination process, so a wider context or reference position is required to determine the correct start of the CDR3. Noncanonical C-terminal motifs (e.g. FXXG or XGXG) also exist. In mouse and man, the conserved motif lies at the −11 and −10 positions relative to the end of the J gene for TRAJ and TRBJ, respectively [49], allowing identification of noncanonical sequences either from irregular J genes or from partial motif deletion during recombination. Once the CDR3 has been identified, productive rearrangements likely to give rise to an expressed TCR chain are usually defined as those which contain a CDR3 sequence, are in-frame with respect to the start of the V gene leader sequence and the end of the constant region and do not contain any premature stop codons. Translation of CDR3s, and identification of productive and nonproductive TCRs, is provided by all the packages discussed above.

## Sequence and abundance error-correction strategies

Errors in nucleotide sequence, where the final data do not faithfully reflect the input molecules, are produced at various stages of library preparation. Such errors occur primarily as a result of either failure of enzymes (typically reverse transcriptase and DNA polymerase) to incorporate the correct nucleotide, or from the wrong base being called during the DNA sequencing reaction itself. These errors are unavoidable: even with fidelities in the order of 1 error per million bases that modern polymerases boast [50, 51], a 20-cycle PCR amplifying a short 300 bp amplicon from 1000 input molecules would be expected to incorporate over 300 000 erroneous bases.

Such errors present a particularly acute problem for analysis of HTS repertoires, as most TCRs only occur once or twice in a sample, and may genuinely differ from another by only single-base differences. Erroneous sequences can therefore be mistaken for genuine sequences and artificially inflate the diversity of a repertoire. As an example, we have sequenced a T-cell clone specific for tetanus toxoid [52] and observed >150 alpha gene and >200 beta gene variant sequences, of which many were present only once in an experiment.

The standard FASTQ output provides a quality (Phred) score for each nucleotide, which estimates the likelihood that the base is called incorrectly. Perhaps the most straightforward error-reduction strategy involves filtering reads or trimming bases with low-quality scores, typically using a threshold score of Q30 [53, 54], which on the Phred score is equivalent to an estimated probability of a base call being incorrect of 1 in 1000 [55]. This technique will only remove errors produced from mistakes during base calling, as PCR errors would not be any more likely to suffer from low quality. Additionally, as quality estimation is influenced by library preparation, sequencing platform and DNA sequence context [56, 57] unsupervised quality filtration can potentially bias removal of particular TCR rearrangements over others [36].

A related approach involves simply removing all low-frequency sequences, as they are those most likely to be produced as a result of errors. This threshold can be fixed *a priori*, e.g. requiring a minimum of five reads per TCR [58] or the threshold can be estimated from the data. In their seminal paper sequencing human TCRβ chains [53], Warren *et al.* reported that without any filtering, there were seemingly thousands of novel J genes detectable in their beta-chain rearrangements; by retaining only the top 96% of reads (‘D96 cutoff’), artifactual sequences were removed, and this number was reduced down to the expected 13 TRBJ genes. However, Nguyen *et al.* [54] reported that the threshold required to reduce repertoires derived from monoclonal TCR transgenic mice to single TCRs differed between experiments. A major disadvantage of this approach, apart from the arbitrary cutoff, is that it undoubtedly removes large numbers of genuine but rare sequences from the data set.

A more sophisticated error editing of HTS TCR sequence data uses clustering to identify similar sets of sequences, and then absorbing the rarer members of each cluster into the more common. An early repertoire paper performed clustering using a ‘nearest neighbour’ algorithm to collapse their short-read TCR data (54 bp) into clusters of sequences, which differed by up to 2 bp (i.e. a Hamming distance ≤2) [5]. The clustering/merging step can be refined by merging lower abundance TCRs into those that are a certain amount higher within some threshold [25] or only doing so if the mismatches are within the V(D)J germline sequences. This retains a greater proportion of the initial TCR diversity [59], but may still risk removal of genuine but rare TCRs, prompting some developers to omit all such steps in favor of retention of the many infrequent sequences [26]. Some pipelines (e.g. MiTCR/MiXCR) allow users to alter thresholds and clustering parameters to favor either removing errors or retaining diversity [22]. Frequency filtering alone cannot detect reverse transcriptase or errors in the early cycles of PCR, as such mistakes will be amplified along with the original sequences. However, several increasingly sophisticated error-correction algorithms based on different statistical models of the data generating process have been published [22, 25, 27, 38], which claim almost error-free profiling of TCR sequences.

**Figure 3:**
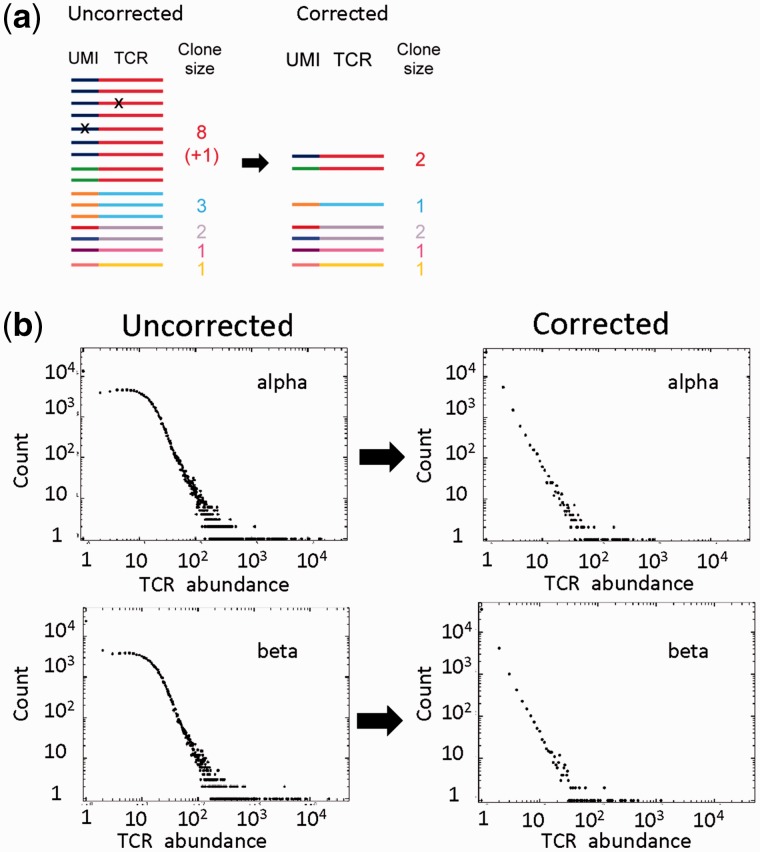
Error correction using UMIs. (**A**) Schematic of the error-correction process. Each TCR is associated with a UMI, which acts as a molecular barcode. TCRs are clustered based on UMI. Identical TCRs within a cluster (i.e. with the same molecular barcode) are collapsed to a count of 1. Minority variants within a cluster are similarly merged with the majority variant. The number of clusters (i.e. same TCR, different UMI) gives the corrected abundance count for that TCR. Optionally, barcodes within a specified molecular distance of each other (usually 1 or 2 Hamming units) can be clustered together. (**B**) The effects of error correction on sequence abundance data for a set of TCR alpha and beta sequences obtained from a sample of unfractionated peripheral blood. The number of TCRs with each abundance observed is plotted against the abundance itself (labeled TCR abundance), e.g. the leftmost point represents the number of TCRs that occur only once in the sample, the next point the number that occurs twice, etc. The figure shows the distribution obtained before (left) and after (right) error correction using UMIs.

A major advance in error correction has come from the incorporation of UMIs (Figure 3). UMIs are short stretches of random nucleotides (sometimes referred to as molecular barcodes) incorporated before PCR amplification, typically during or immediately after the reverse transcription. After PCR, UMIs can be used to identify which sequences derived from the same single starting mRNA molecule, as they would all have been tagged with the same UMI and, thus, can be counted together. This information can be used for two distinct, although related, processing objectives. The first is to identify those duplicate sequences that are derived by PCR amplification from the same initial template molecule. This is crucial to obtaining accurate information on TCR abundance within a mixed repertoire. The second is to identify and correct for sequence errors introduced during PCR or the sequencing protocol itself.

A protocol incorporating UMIs was used in the first paper to use HTS to sequence adaptive immune repertoires [60]. Although UMIs were subsequently also incorporated into general quantitative RNA-seq techniques [61–63], UMIs were not commonly used in TCR repertoire studies until more recently [15, 16, 64]. The introduction of UMIs allows much more powerful error profiling algorithms to be deployed. Current protocols such as MiGEC [64] and the latest version of Decombinator use 12-nucleotide UMIs, which provides a diversity of 4^12^ (>16 × 10^6^) different barcodes, likely to far exceed the number of TCR cDNA molecules in a sample. During analysis, sequences are first clustered based on common UMIs. Sequences within such groups are then compared; as errors are relatively rare, the infrequent but similar sequences within a cluster are highly likely to be derived from the more common, and so minority species that differ by a small number of mismatches are absorbed into the majority variant. After removing erroneous TCRs by collapsing on UMIs, the UMIs themselves associated with each TCR can be error corrected (e.g. by clustering using Hamming distance) and counted to give a corrected abundance of that sequence and the number of original starting molecules in the sample. This correction is especially important as PCR is intrinsically a stochastic process, and amplification rates of individual molecules may vary depending on the amplified sequence, the primer sequences and the reaction conditions. Even using identical starting materials, PCR efficiencies within a single reaction can vary widely [65].

UMI strategies therefore offer a way to robustly correct both the qualitative and quantitative parameters of repertoire data (Figure 3), providing the only means to infer actual numbers of molecules [61]. Some technical limitations remain. Biases in the production of ‘random’ sequences in synthetic oligonucleotides [66] probably account for biases observed in previous TCR studies using UMIs [67]. Such biases likely reduce the actual potential size of the UMI pool below the theoretical maximum. Another drawback of current barcoding protocols is that the UMIs are incorporated at the opposite end of the amplicons to the constant region [15, 16]. This results in UMIs being sequenced in the reverse read (Read 2) of a paired-end Illumina sequencing run, which typically has lower base qualities and a higher probability of error than the forward read (Read 1) [55].

Several pipelines incorporate a combination of threshold, clustering and UMI-based error-correction capabilities (e.g. MiXCR followed by MIGEC). A recent tool, RTCR, is of particular note for taking a ‘data-driven’ approach to TCR error correction, in that quality and frequency thresholds are inferred from fitting the data to statistical models, which are used for the analysis [27]. RTCR performs a number of sophisticated clustering processes, after estimating the error rate for each sample by counting mismatches between germline regions and reference sequences. To try and prevent clustering of genuine but related TCRs, RTCR also estimates expected frequencies of each TCR, and prevents merging of clusters if the resultant clusters would exceed the expected.

## Benchmarking and its challenges

New tools for TCR gene assignment and error correction are frequently accompanied by benchmarking, which seeks to compare their performance to other programs. However, benchmarking on real data sets is not straightforward, as we cannot know the ‘true’ repertoire of a sample before we have measured it. Generating simulated repertoire data is a powerful method and widely adopted, but is far ‘cleaner’ than actual data will be. It also incorporates assumptions, which may not always be true. For instance, simulated PCR or sequencing processes often assume a uniform distribution of errors along the sequence [20, 25, 27, 36, 68]. The reality is that sequence motif-dependent error profiles have often been described [55, 69–71]. MiTCR refines this assumption somewhat by learning substitution probabilities from high-quality germline regions [38], but there is room for further improvement in this area. A related problem is that of TCR polymorphism: while possessing less allelic diversity than the immunoglobulin chain loci, there are TCR genes with multiple alleles and likely many with undiscovered ones. Such alleles could easily be misconstrued as errors, or even result in V or J regions being misclassified. Some HTS repertoire studies have reported additional V and J regions [25, 72]; ideally, such discoveries should be fed back into databases such as IMGT, so that they can be included in subsequent analyses.

In conclusion, gene assignment and error correction for HTS of TCRs provide special challenges, which have been addressed in a number of ways (Table 1). Some of the most commonly used tools have been outlined above. In addition, commercial providers of TCR sequencing (Adaptive Biotechnology, iRepertoire) generally provide gene assignments as part of the output provided. As these are not open source, they are not discussed in detail here, but users with little bioinformatics or computational skills may opt to use this output directly for high-level processing.

The bioinformatics approaches outlined above differ in ease of implementation, accuracy, speed and coverage, often performing optimally when used in conjunction with the experimental protocol for which they were initially developed. All the tools discussed provide high-quality output suitable for most downstream analysis currently used (see sections below). No optimal state-of-the-art analysis pipeline has emerged so far, and the choice of tool will depend on the computational skill set of the user and the data sets to be used.

## High-level repertoire processing: revealing biological and clinical meaning

The discussion so far has centered on methodological issues, which arise in processing high-throughput TCR sequence data and extracting from it a quantitative catalog of individual TCR frequencies within a population of T-cells. This catalog constitutes the T-cell repertoire of a sample. This ‘preprocessing’ has generated an active literature, as reviewed in the sections above, and has produced some interesting solutions to some difficult computational and bioinformatics questions. However, biological insight into the underlying immunology necessarily requires higher-level analysis. Some of the approaches and challenges raised by this analysis are discussed below.

The enormous diversity of the TCR repertoire results in individual experiments capturing thousands or even millions of different sequences from a single sample [73]. Furthermore, two different samples, even if taken from the same individual, often only have a small degree of overlap. It is not obvious how to extract information from data of such diversity and heterogeneity. A major focus of much of the TCR repertoire analysis to date has therefore been the analysis of summary statistics, which can capture some of the essential information about a repertoire in a small number of parameters. These include comparative V and J region usage, which provides an expanded version of older antibody- or PCR-based techniques [74–78]. Unexpectedly, V and J gene usage turn out to be highly non-uniform, following an underlying pattern that is remarkably conserved across different individuals and may reflect transcriptional regulation encoded at the level of chromatin remodeling [79] or biases in the DNA recombination process [80]. Other parameters include CDR3 length distribution, measures of diversity and measures of overlap between repertoires. These metrics are mostly rather straightforward to compute, and algorithms and packages are available in many computer languages, including R and Python. However, a number of specialized toolsets have been published that aim to facilitate the process of analysis and to provide summary plots and statistics without requiring significant computational skills. These include VDJtools [81], TCR (an R package for T-cell repertoire analysis) [82] and the ‘in house’ analysis tool which accompanies Adaptive Biotechnology’s ImmunoSeq pipeline (www.adaptivebiotech.com/immunoseq/analyzer). Typically, these tools provide summary statistics on individual sequencing runs, including how many total, unique, productive and nonproductive sequences were found. The proportional usage of each V(D)J gene, and diversity metrics are also frequently provided. Most tools also allow comparison between two or more sequence collections, measuring differential abundance of TCRs, and differential V(D)J usage, which can be quantified using the Jensen Shannon divergence index [15].

VDJtools and TCR are open source projects, which theoretically allow for further user-driven modification and development. However, neither VDJtools nor TCR provide Web browser interfaces, and the need to use R scripting or command line Java is a significant barrier to entry for researchers without computational experience. The newly released programs SeeTCR (http://friedmanlab.weizmann.ac.il/SeeTCR/) and ARResT/Interrogate [83], which combine the statistical power of R with a user-friendly Web browser interface via use of the Shiny package, may provide a good alternative for the growing community of people interested in T-cell repertoire analysis but with only basic computational skills. The recently released IMGT/StatClonotype R package similarly offers gene-level comparison metrics and tools with which to compare pairwise repertoire samples via a graphic user interface [84], including calculation of various diversity metrics, which describe TCR sequence frequency distributions (see below).

One specific application of repertoire analysis focuses on tracking the abundance of specific TCR rearrangements over time in longitudinal data from the same individual. This has found particular application in monitoring ‘minimum residual disease’ following treatment of B-cell or T-cell malignancies [85–87], as the abundance of a disease-associated sequence can then be used to manage patient care and provide early diagnosis of remission. The TCR and BCR rearrangements in such cancers are often incomplete and nonproductive. VIDJIL [21] provides an open source Web-based portal specifically (although not exclusively) developed for the identification of such clones and following them over time [87]. As sequencing costs fall, and experimental pipelines become cheaper and more robust, the ability to provide graphical, easily interpreted and informative summaries of repertoire data is likely to become the key to incorporating repertoire measurements into routine clinical practice.

A powerful statistical conceptual framework in which to consider the TCR repertoire has been provided by the work of Thierry Mora and Aleksandra Walczack [72, 88]. The model uses hidden Markov models to assign a probability to each sequence within a repertoire. The parameters of the model can be learnt from data, using productive or nonproductive sequences. Such models can provide quantitative parameters describing the multiple generative and selective processes that give rise to repertoires [89]. For example, the model suggests that public clones (those sequences found in many individuals within a population) arise, at least in part, because they are generated with greater frequency by the stochastic TCR recombination process than private ones (sequences found in only one individual).

## Measures of diversity

**Figure 4:**
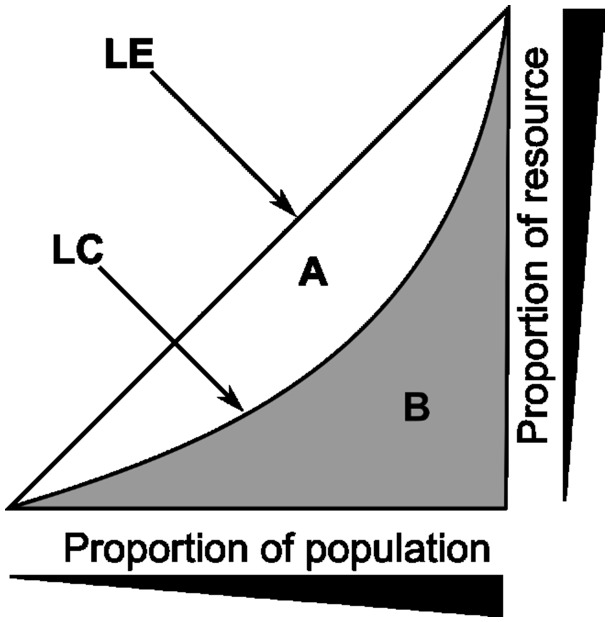
The Lorenz plot and the Gini index. LE – line of equality; LC – Lorenz curve; the Gini index is defined as the ratio of the areas A/(A + B) (0≤G≤1). Individual members within a population, which in the context of the repertoire is unique TCR sequences, are ranked in order of abundance. The Lorenz curve is obtained by plotting the cumulative abundance of each TCR against its rank (lowest to highest). If all individual species are of equal abundance, the Lorenz curve follows the diagonal, and the Gini index is zero. The more unequal the distribution of abundances, the larger the Gini index (≤1).

**Figure 5:**
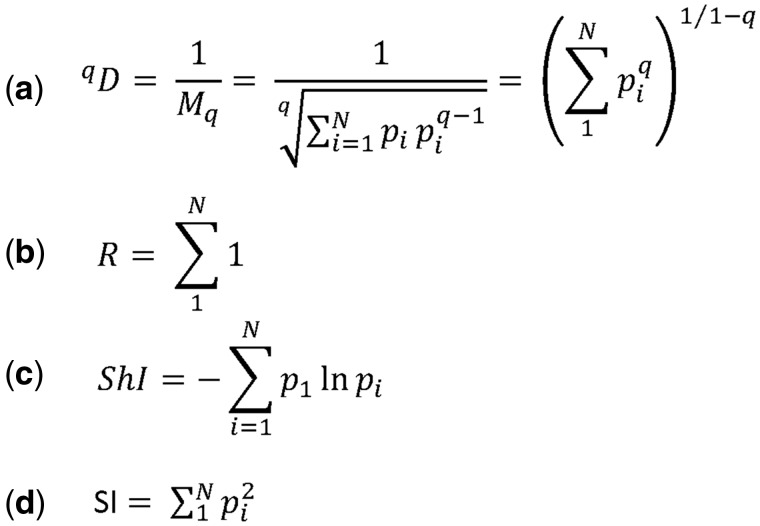
Measures of diversity. (**A**) The ‘true diversity’ refers to the number of equally abundant species within a population needed for the average proportional abundance of the species to equal that observed (where all species may not be equally abundant). It is calculated as the inverse of the weighted generalized mean of order *q* of the proportion (p) of each species within a population of size *N*. (**B**) The richness (*R*) is the number of distinct species observed. It is equivalent to ‘true diversity’ of order 0. (**C**) Shannon diversity index (ShI, often referred to as the Shannon Entropy) is the logarithm of the ‘true diversity’, as q → 1. (**D**) The Simpsons diversity index (SI) is the inverse of the ‘true diversity’ of order 2.

Among all the summary statistics arising from repertoire data, the measurement of diversity has perhaps generated the most discussion [90]. The concept of diversity itself combines two distinct metrics, the total number of different sequences within a sample and their frequency distribution. The former is most directly captured by species richness, a term borrowed from the ecological literature, while the latter is captured by the Gini inequality index (Figure 4), used widely in economics. A large number of other measures have been developed, which seek to capture a combination of richness and inequality [91]. All these measures are related to the general series of ‘true diversity measures’ (Figure 5) [92], a set of weighted generalized means of the proportion of each species in a population. The Simpson index (true diversity of order 2) is one of the most often used. The Shannon index, another commonly reported number, corresponds to the logarithm of the true diversity as *q*, which defines the order of the diversity measure (Figure 5) tends to 1 (the diversity index is undefined when *q* = 1).

An additional complication in interpreting measurements of diversity is that the measures are strongly influenced by the large numbers of rare species (often present only once) that are typically observed in a repertoire sample, and which are themselves dependent on sequencing error and the accuracy of the algorithms used to correct this (see above). In view of the relatively small proportion of the total T-cell population that can be sampled (typically 1 in 10^4^ or less for human blood samples) and residual uncertainty over sequencing error rates even after correction, estimates of the total diversity or richness of the human immune system need to be treated with caution. Despite these reservations, the relative TCR repertoire diversity within populations may still provide useful information on disease status [93, 94].

Another interesting, but still not well understood, phenomenon is the existence of public and private TCRs or CDR3 sequences [95–98]. The definition of these terms is not rigorous, but typically private sequences are those found in only one, or at most a small proportion of repertoires taken from different individuals. There is general agreement that such private sequences constitute the vast majority of TCRs. In contrast, a smaller proportion of TCR sequences are found in a large majority of repertoires taken from different individuals. Public clones have been reported in a variety of antigen-specific responses, even from individuals of different MHC haplotypes. The biological significance of public clones remains unclear, although they have been associated with diverse functions from regulatory self-immunity [98] to control of EBV [99] and HIV [100] infection.

## Antigen specificity

We finish by briefly considering the most challenging of tasks confronting TCR repertoire analysis, namely, relating the sequences now available in public databases in their billions, to their potential antigen specificity. This challenge is compounded by the fact that individual TCRs are likely to recognize many different antigens (degeneracy) and that many different TCRs may recognize the same antigen [101–103]. We consider two approaches exemplified in the recent literature. The first approach is to generate databases of TCRs derived from cells of known specificity, for example from MHC multimer sorting, or *in vitro* restimulation/cloning. Two such open access sequence databases are available for download (https://vdjdb.cdr3.net and http://friedmanlab.weizmann.ac.il/McPAS-TCR/), although detailed descriptions have not yet been published. Both list sets of annotated sequences drawn from the literature (surprisingly using largely non-overlapping data sources). The databases can be used either to extract sets of TCRs specific for particular antigens or to search for sets of antigen-specific TCRs within new data sets. The databases contain in the order of a few thousand sequences, and this number is likely to grow as more powerful techniques for obtaining paired sets of TCR αβ genes develop. Such databases are likely to be biased toward public TCRs, which will be more often represented in individual data sets. Nevertheless, as the databases grow, they may provide valuable diagnostics or prognostic tools not just with respect to infectious diseases but also cancer, autoimmunity and others [7]. However, given the size of the potential T-cell repertoire, which even conservative estimates put at hundreds of millions of sequences, such databases are only likely to capture a small fraction of the total.

A second complimentary approach is to try and derive some general rules that can be used to map the T-cell receptor sequence to antigen specificity. A mechanistic approach, based on predicting structure alone, is likely to be challenging. Frequently, only one chain of the TCR is known. Furthermore, the CDR3 region of the TCR adopts an unstructured loop formation, which makes precise structural predictions hard. Finally, even if the three-dimensional structure could be predicted accurately, predicting the binding target is likely to be hard as the interaction is of low affinity and distributed over several residues [104]. An example of an alternative machine learning-based approach is described in [105]. In this study, a probabilistic model of T-cell recognition emerges, in which antigen specificity is considered as an emergent global property of the repertoire as a whole. Specific antigen recognition can be predicted by the frequency of short stretches of amino acids within the CDR3 sequence. Many of these motifs are found at the N- or C-terminal of the CDR3 region, perhaps, offering an explanation of the well-established bias in V and J region usage, which has often been observed in populations of antigen-specific T-cells [100, 106].

## General conclusion

The first decade of TCR HTS has been focused on overcoming the technical problems associated with parallel short-read sequencing of such a diverse set of DNA molecules. Improvements in the sequencing technology, combined with the development of increasingly sophisticated bioinformatics analysis tools, can now provide fast and almost error-free TCR repertoires. In contrast, high-level processing remains relatively underdeveloped. A number of tools exist that provide basic parameters of the repertoire, such as diversity and V(D)J usage, allowing basic comparisons between different samples. However, the major challenge remains linking the TCR sequences to function, and specifically to antigen specificity. Sophisticated machine learning algorithms are being developed that can combine the paradoxical degeneracy and cross-reactivity of individual T-cell receptors with the specificity of the overall T-cell immune response. Such computational analysis will provide the key to unlock the potential of the T-cell receptor repertoire to give insight into the fundamental biology of the adaptive immune system and to provide powerful biomarkers of disease.

## Notes

**James Heather** is a research fellow working on T-cell receptor repertoires at Massachusetts General Hospital in Boston, having recently moved from the Innate2Adaptive research group (http://www.innate2adaptive.com/). He previously completed his doctoral thesis on a study of the T-cell repertoire in chronic HIV under the supervision of Benny Chain.

## Notes

**Mazlina Ismail** is a bioinformatics graduate student under the supervision of Benny Chain. Her thesis is focused on analysis of T-cell receptor repertoires in health and disease.

## Notes

**Theres Oakes** is a postdoctoral research associate studying the T-cell receptor repertoire in the context of skin contact hypersensitivity. She completed her doctoral thesis on the interaction between dendritic cells and keratinocytes in skin allergy/sensitization under the supervision of Benny Chain.

## Notes

**Benny Chain** is Professor of Immunology in the Division of Infection and Immunity at UCL, and codirector of the Innate2Adaptive research group. His research focuses on computational and systems approaches to the study of immunology, especially in the context of the T-cell receptor repertoire.
